# The Link Between COVID-19, Anxiety, and Religious Beliefs in the United States and the United Kingdom

**DOI:** 10.1007/s10943-021-01296-5

**Published:** 2021-05-29

**Authors:** Francesco Rigoli

**Affiliations:** Department of Psychology, City, University of London, Northampton Square, London, EC1V 0HB UK

**Keywords:** Anxiety, Stress, Religion, Coronavirus, COVID-19, Controllability

## Abstract

Research has shown that stress impacts on people’s religious beliefs. However, several aspects of this effect remain poorly understood, for example regarding the role of prior religiosity and stress-induced anxiety. This paper explores these aspects in the context of the recent coronavirus pandemic (COVID-19). The latter has impacted dramatically on many people’s well-being; hence it can be considered a highly stressful event. Through online questionnaires administered to UK (*n* = 140) and USA (*n* = 140) citizens professing either Christian faith or no religion, this paper examines the impact of the coronavirus crisis upon common people’s religious beliefs. Anxiety about the coronavirus and prior religiosity showed an interaction effect upon change in religious beliefs (*t*(276) = 2.27, *p* = .024): for strong believers higher anxiety about coronavirus was associated with increased strengthening of religious beliefs (*r* = .249), while for non-believers higher anxiety about coronavirus was associated with increased scepticism towards religious beliefs (*r* = − .157). These observations are consistent with the notion that stress-induced anxiety enhances support for an individual’s existing ideology already embraced before a stressful event occurs. This study sheds light on the psychological and cultural implications of the coronavirus crisis, which represents one of the most serious health emergencies in recent times.

## Introduction

Confidence in religious beliefs varies substantially across people, with some individuals reporting strong religion views, others endorsing religion only weakly, and with another group of people rejecting religious claims altogether (Aldridge, 2007; Burchardt et al., 2015). Furthermore, the strength of religious beliefs often fluctuates throughout the life of single individuals (Kimble & McFadden, 2002; Rambo, 1999). Hence a fundamental question is which factors affect the strength of religious beliefs. Stress has emerged as one of such factors (Graham et al., 2001; Pargament, 2001; Park, 2005). Research has observed that, when experiencing distressing situations, some people manifest increased religious conviction (Atran, 2002; Barrett, 2004; Gill & Lundsgaarde, 2004; Sibley & Bulbulia, 2012). These studies have raised the possibility that increasing religious commitment represents a strategy to cope with stress (Pargament, 2001; Pargament & Raiya, 2007). Contrary to early theories interpreting religion as a dysfunctional coping strategy (Hewitt, 2014), empirical evidence suggests that religion often helps believers in dealing with stress successfully, especially by regulating their affective state (Pargament, 2001; Pargament & Raiya, 2007). Religion might be effective in managing stress in as much as it can offer an explanation for stressful events, a source of hope, and a sense of belonging to a religious community (Pargament, 2001; Pargament & Raiya, 2007). However, important aspects of the relationship between stress and religion remain to be fully understood. First, are all individuals equally likely to strengthen their religious beliefs in response to stress? Or can we identify certain characteristics that predispose people to do so? An obvious candidate is represented by people’s prior religious conviction, reflecting how strong religious beliefs are before a stressful event occurs. Different hypotheses can be made about this factor. When experiencing stress, individuals with stronger prior religious beliefs might enhance these beliefs more than people with weaker prior religious beliefs, but the opposite might also occur. A related question concerns the impact upon non-religious people. For them, stress might have no impact on religious beliefs, it might render religion more attractive, or it might decrease the appeal of religion even further.

Another open question concerns the factors mediating the relationship between stress and religious beliefs. One possibility arises out of the proposal that religion is bolstered by perceiving lack of control of the own life (Kay et al., 2010; Norris & Inglehart, 2004). In this perspective, religion would emerge as a compensatory mechanism aimed at re-establishing control. This implies that feeling a loss of control might mediate the effect of stress on religious beliefs. Anxiety, especially when elicited by death awareness, is another potential variable modulating the effect of stress on religion (Jong et al., 2012, 2018; Norenzayan & Hansen, 2006; Vail et al., 2010, 2012). A possibility is that anxiety bolsters faith only in religious individuals (Jong et al., 2012; Norenzayan & Hansen, 2006). Alternatively, anxiety might render religious beliefs more attractive for both believers and non-believers (Vail et al, 2012; Willer, 2009).

The present paper explores the impact of stress upon religious beliefs by examining the role of prior religiosity, the influence upon non-religious persons, and the role of perceived controllability and anxiety. The paper explores these aspects in the context of the ongoing COVID-19 pandemic (Wang et al., 2020). This is due to the spread of a new type of virus attacking the human respiratory system characterised by high levels of infectivity and mortality (Wang et al., 2020). In response to the increasing number of infected patients, many countries have adopted unprecedented policy measures such as the closure of economic activities and the prescription to stay home for citizens. Debate about the coronavirus has monopolised the media and the public discourse, leading to widespread anxiety even for those less directly affected. Given its dramatic consequences for the physical and psychological well-being, the coronavirus pandemic represents a severe cause of stress for many across the globe. Through collection of on-line questionnaire data, this study explores how the coronavirus pandemic has affected common people’s religious beliefs within the USA and the UK, with a focus on Christian and non-religious individuals.

Scholars have started to explore the role of religion in the context of the COVID-19 pandemic (presented in two journal special issues: Carey et al., 2020; Hart & Koenig, 2020). However, the questions identified above remain to be examined. First, have believers and non-believers changed their attitude towards religion after the pandemic outbreak? Second, has coronavirus-related anxiety impacted on religious beliefs of believers and non-believers? This paper focuses on these questions.

## Methods

### Participants

Recruitment of participants was carried out online using the Prolific website (www.prolific.co). Any (18 years old or older) individual from any country interested in participating to online social science studies can register with the Prolific website. Individuals receive monetary reward after participating to a study. Most people get to know Prolific via social media, poster/flyer campaigns at universities, and through referrals from researchers and participants already using the site. When registering to Prolific, individuals are asked demographic questions which later allow researchers to prescreen participants during recruitment. When a researcher creates a new study, any eligible participant (i.e., those meeting the prescreening criteria) can sign in and participate until the sample in complete (the sample size is established a priori). Eligible participants are informed that a new study is available because the study becomes visible to them when accessing the Prolific website, and because the Prolific system sends an email to a random subset of eligible participants.

For the present study, 280 adults were recruited, half females and the other half males, and half UK citizens and the other half USA citizens (the number of participants for each gender and country condition was established a priori). Participants were initially pre-screened (using the Prolific pre-screening) so that they reported either Christian faith or absence of religious faith. All participants were tested on the 30th March 2020. The study was approved by the Research Ethics Committee of the University supporting the study (located in the UK).

### Measures and Procedures

Through the online platform Prolific, participants answered on-line questions assessing:

- Religiosity, measured by a Likert-type item asking “How religious are you?” (1 = not at all religious, 2 = a little religious, 3 = moderately religious, 4 = quite religious, 5 = very religious).
- Change of religious beliefs due to the coronavirus outbreak (${Religion}_{COVID}$), measured by a Likert-type item asking “Since coronavirus emergency started, your religious beliefs have” (1 = become substantially weaker, 2 = become a little weaker, 3 = remained the same, 4 = become a little stronger, 5 = become substantially stronger).
- Personal feeling of control associated with the coronavirus pandemic ($${Contr}_{PERS})$$, measured by a Likert-type item asking “How much do you feel able to manage the coronavirus emergency at the personal level, namely with regard to you and your family?” (1 = not at all, 2 = slightly, 3 = moderately, 4 = considerably, 5 = extremely).
- Control attributed to authorities with regard to the coronavirus pandemic ($${Contr}_{AUTH})$$, measured by a Likert-type item asking “How much do you trust authorities in their ability to manage the coronavirus crisis?” (1 = not at all, 2 = slightly, 3 = moderately, 4 = considerably, 5 = extremely).
- Anxiety elicited by the coronavirus crisis ($${Anx}_{COVID})$$, measured by a Likert-type item asking “Do you feel more anxious because of the coronavirus crisis?” (1 = not at all, 2 = slightly, 3 = moderately, 4 = considerably, 5 = extremely).
- Neuroticism, measured by the associated 8-items scale taken from the BIG 5 personality Questionnaire (John & Srivastava, 1999). The scale includes statements such as “I am depressed, blue” that are evaluated on a Likert scale (1 = strongly disagree, 2 = disagree, 3 = neither agree nor disagree, 4 = agree, 5 = strongly agree).
- Intolerance of uncertainty (IoU), measured by a 12-items scale previously validated (Buhr & Dugas, 2002). The scale includes statements such as “unforeseen events upset me greatly” that are evaluated on a Likert scale (1 = strongly disagree, 2 = disagree, 3 = slightly disagree, 4 = neutral, 5 = slightly agree, 6 = agree, 7 = strongly agree).

Before answering these questions, participants completed a demographic questionnaire assessing gender (through the question “Indicate you gender”, with male and female as options), age (therough the question “Indicate you age”, to which participants answered writing the corresponding number), and country (through the question “Indicate you country”, with UK and USA as options). Filling the questionnaires took approximately 3 min, and subjects were paid £0.25 for participating.

### Statistical Analysis

First, we calculated the mean and SD of the variables. Next, we performed statistical hypothesis testing analyses, in all cases adopting two-tailed *p* < 0.05 as significance threshold. Tests included Pearson correlation, one- and two-sample t-tests, and multiple regression. IBM SPSS was adopted for analysing data.

## Results

**Fig. 1:**
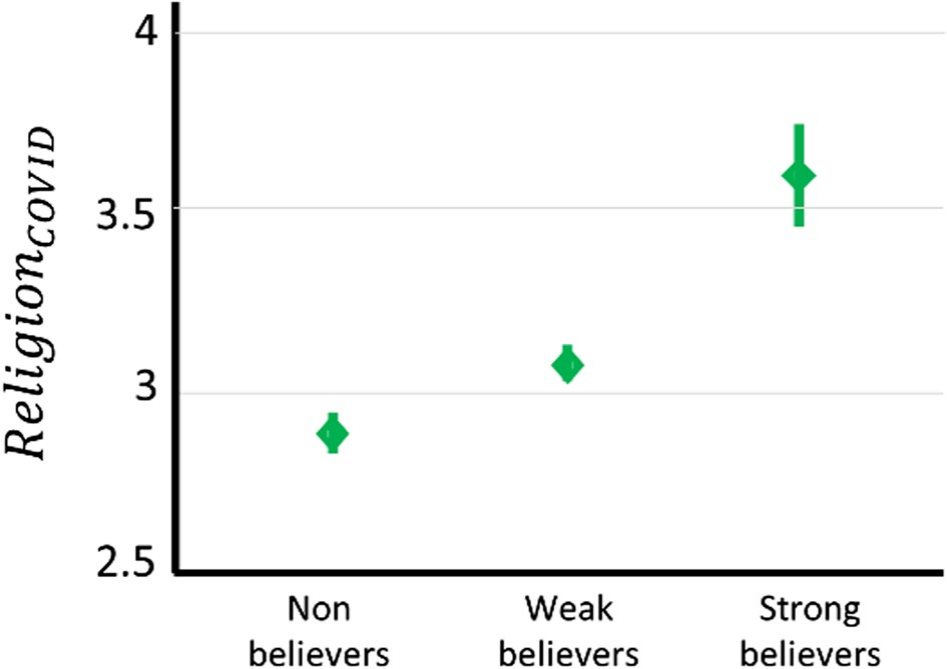
Reported change in strength of religious beliefs following the coronavirus crisis ($${Religion}_{COVID})$$. Data are described for three different groups of participants varying with respect to their level of Religiosity, namely for non-believers, weak believers, and strong believers

Descriptive statistics are reported in Table 1 (for categorical variables) and Table 2 (for continuous variables), and Pearson correlations among variables are reported in Table 3. Our first question was whether the level of religiosity was related with self-reported change in the strength of religious beliefs in response to the coronavirus crisis (${Religion}_{COVID}$). We observed a significant positive correlation (Fig. 1; *r*(278) = 0.393, *p* < 0.001). To ascertain that this was independent of country, we repeated the analysis separately for each country, and found a significant correlation in both the USA (*r*(138) = 0.453, *p* < 0.001) and the UK (*r*(138) = 0.322, *p* < 0.001). To probe further the correlation between Religiosity and ${Religion}_{COVID}$, we arranged participants in three groups based on their religiosity: non-believers (those reporting to be not at all religious) (*n* = 147), weak believers (those reporting to be a little or moderately religious) (*n* = 98), and strong believers (those reporting to be quite or very religious) (*n* = 35). A one-way ANOVA comparing groups with respect to ${Religion}_{COVID}$ indicated a significant effect (Fig. 1; *F*(2,277) = 24.60, *p* < 0.001). Binary comparisons revealed that strong believers compared to weak believers strengthened more their religious beliefs in response to the coronavirus pandemic (*t*(243) = 2.99, *p* = 0.003), while weak believers strengthened their religious beliefs more than non-believers (*t*(131) = 4.78, *p* < 0.001).

**Table 1:** Categorical variables measured in the study

Categorical variables	*n*	%
**Gender**		
Female	140	50
Male	140	50
**Country**		
UK	140	50
USA	140	50

**Table 2:** Continuous variables measured in the study

	Minimum	Maximum	Mean	SD
Religiosity	1	5	1.92	1.20
${Anx}_{COVID}$	1	5	3.43	1.12
${Contr}_{AUTH}$	1	5	2.67	.98
${Contr}_{PERS}$	1	5	3.29	.87
${Religion}_{COVID}$	1	5	3.04	.59
IoU	19	84	53.07	11.52
Neuroticism	9	36	4.57	5.59
Age	18	71	34.8	11.71

**Table 3:** Pearson correlation (and associated p value) among the variables measured in the study

	${Anx}_{COVID}$	${Contr}_{AUTH}$	${Contr}_{PERS}$	${Religion}_{COVID}$	IoU	Neuroticism	Age	Gender
Religiosity	*r* = .058 *p* = .333	*r* = 0.104 *p* = .081	*r* = .084 *p* = .159	*r* = .393** *p* < .001	*r* = − .067 *p* = .261	*r* = − .113 *p* = .060	*r* = .221** *p* < .001	*r* = − .039 *p* = .519
${Anx}_{COVID}$		*r* = .010 *p* = .866	*r* = − .189** *p* = .001	*r* = − .011 *p* = .849	*r* = .397** *p* < .001	*r* = .423** *p* < .001	*r* = .067 *p* = .265	*r* = .202** *p* = .001
${Contr}_{AUTH}$			*r* = .287** *p* < .001	*r* = .067 *p* = .260	*r* = − .082 *p* = .169	*r* = − .103 *p* = .086	*r* = .121* *p* = .043	*r* = .065 *p* = .275
${Contr}_{PERS}$				*r* = .059 *p* = .322	*r* = − .226** *p* < .001	*r* = − .232** *p* < .001	*r* = .072 *p* = .227	*r* = − .037 *p* = .538
${Religion}_{COVID}$					*r* = − .073 *p* = .223	*r* = − .014 *p* = .816	*r* = .071 *p* = .239	*r* = .048 *p* = .420
IoU						*r* = .587** *p* < .001	*r* = .001 *p* = .988	*r* = − .005 *p* = .934
Neuroticism							*r* = − .115 *p* = .055	*r* = .056 *p* = .347
Age								*r* = .181** *p* = .002

*p < .05; **p < .001

This analysis tells us that there is a difference among non-believers, weak believers, and strong believers. However, it does not tell us whether, in response to the coronavirus crisis, these groups strengthened or weakened their religious beliefs. To address this question, we ran one-sample t-tests for each group with regard to ${Religion}_{COVID}$, testing the null hypothesis that the average score was equal to 3 (note that the score of 3 describes religious beliefs that remained unchanged). These analyses revealed that, after the coronavirus pandemic, strong believers strengthened their religious beliefs (Fig. 1; *t*(34) = 4.58, *p* < 0.001), weak believers left their beliefs unchanged (Fig. 1; *t*(97) = 1.81, *p* = 0.073; note this result reflects a non-significant trend towards strengthening religious beliefs), and non-believers weakened their religious beliefs (Fig. 1; *t*(146) = − 2.58, *p* = 0.011).

Next, we asked whether feeling of control mediates the effect of stress on the strength of religious beliefs (Kay et al., 2010; Norris & Inglehart, 2004). In the context of our study, this possibility predicts that the lower the perceived control regarding the coronavirus crisis, the more religious beliefs will be strengthened. We tested this both regarding personal control ($${Contr}_{PERS})$$ and control attributed to authorities ($${Contr}_{AUTH})$$. In both instances, we did not find any relationship with $${Religion}_{COVID}$$ ($${Contr}_{PERS}$$: *r*(278) = 0.059, *p* = 0.322; $${Contr}_{AUTH}$$: *r*(278) = 0.067, *p* = 0.260). For exploratory purposes, we ran the same analyses for each religious group separately (strong believers, weak believers, non-believers) and found no correlation in any case (strong believers: $${Contr}_{PERS}$$: *r*(33) =  − 0.074, *p* = 0.671; $${Contr}_{AUTH}$$: *r*(33) =  − 0.037, *p* = 0.834) (weak believers: $${Contr}_{PERS}$$: *r*(96) =  − 0.078, *p* = 0.444; $${Contr}_{AUTH}$$: *r*(96) =  − 0.003, *p* = 0.977) (non-believers: $${Contr}_{PERS}$$: *r*(145) = 0.038, *p* = 0.650; $${Contr}_{AUTH}$$: *r*(96) = 0.071, *p* = 0.392). To explore this further, we considered the Neuroticism and Intolerance of Uncertainty scales as covariates. We estimated two multiple regression models both having $${Religion}_{COVID}$$, Neuroticism and Intolerance of Uncertainty as predictors. One model had $${Contr}_{PERS}$$ as dependent variable, the other had $${Contr}_{AUTH}$$ as dependent variable. In neither case $${Religion}_{COVID}$$ exerted a significant effect ($${Contr}_{PERS}$$: *t*(276) = 0.82, *p* = 0.415; $${Contr}_{AUTH}$$: *t*(276) = 1.07, *p* = 0.284).

**Fig. 2:**
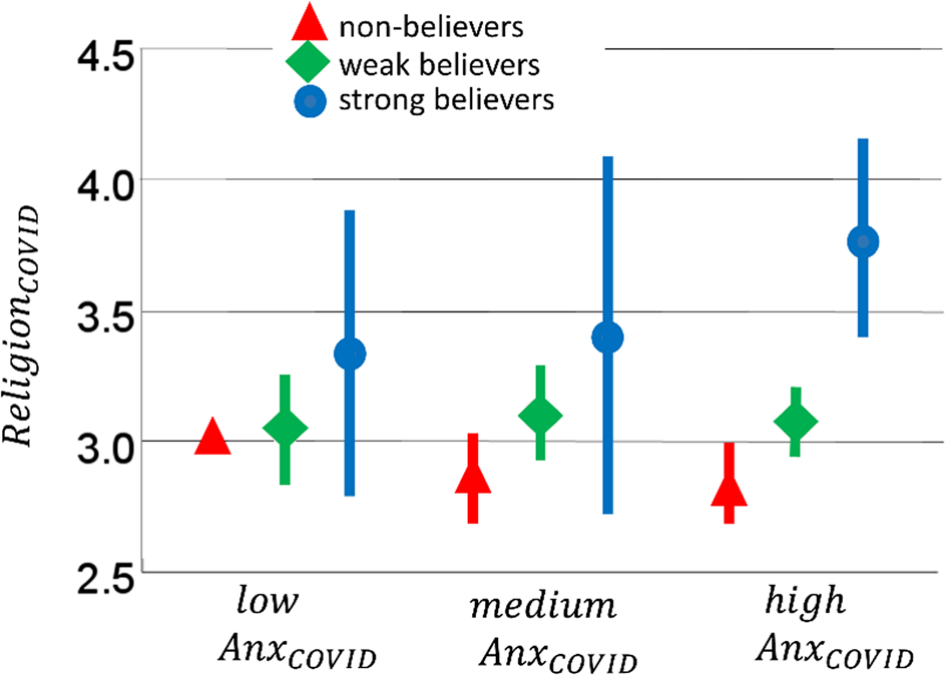
Change in strength of religious beliefs following the coronavirus crisis ($${Religion}_{COVID})$$ as a function of anxiety in response to the coronavirus pandemic ($${Anx}_{COVID})$$. For the latter variable, participants are separated in three groups ($$low {Anx}_{COVID}$$: participants who responded “not at all” or “slightly” to the related question; $$medium {Anx}_{COVID}$$: participants who responded “moderately”; $$high {Anx}_{COVID}$$: participants who responded “considerably” or “extremely”). Different colours describe participants with different level of Religiosity (Color figure online)

Finally, we asked whether the anxiety elicited by the coronavirus pandemic (${Anx}_{COVID}$) had any impact on the change in religious beliefs (${Religion}_{COVID}$). One possibility is that anxiety bolsters religious beliefs for both believers and non-believers (Vail et al, 2012). In the context of our study, this predicts a positive relationship between ${Anx}_{COVID}$ and ${Religion}_{COVID}$. Another possibility is that anxiety leads people to embrace their prior beliefs with enhanced confidence (Jong et al., 2012). In the context of our study, this predicts that prior religiosity will moderate the effect of anxiety in such a way that for strong believers anxiety will bolster religious beliefs and for non-believers anxiety will weaken religious beliefs. To test these predictions, we z-scored ${Anx}_{COVID}$, ${Religion}_{COVID}$ and Religiosity, obtaining ${ZAnx}_{COVID}$, ${ZReligion}_{COVID}$, and ZReligiosity, respectively. Then, we estimated a regression model of ${ZReligion}_{COVID}$ having ${ZAnx}_{COVID}$, ZReligiosity, and their interaction as predictors (note that Religiosity and ${Anx}_{COVID}$ were not correlated: *r*(278) = 0.058, *p* = 0.333). The model explained a substantial proportion of variance (*R*^2^ = 0.17, *F*(3,276) = 18.52, *p* < 0.001). While ${ZAnx}_{COVID}$ did not contribute to the model (*t*(276) =  − 0.35, *p* = 0.724), both ZReligiosity (*t*(276) = 7.01, *p* < 0.001) and the interaction term (*t*(276) = 2.27, *p* = 0.024) did. To interpret the role of the interaction term, we estimated the Pearson correlation between ${Anx}_{COVID}$ and ${Religion}_{COVID}$ separately for the different Religiosity groups and we observed a score of *r* = 0.249, *r* = 0.007, and *r* = − 0.157 for strong believers, weak believers and non-believers, respectively (Fig. 2). This observation fits with the prediction that, in the context of the coronavirus pandemic, prior religiosity moderates the effect of anxiety in such a way that for strong believers anxiety strengthens religious beliefs and for non-believers anxiety weakens religious beliefs (Jong et al., 2012).

## Discussion

This paper explores the impact of the coronavirus pandemic upon common people’s religious beliefs. Given its consequences for physical and mental well-being (Wang et al., 2020), the coronavirus crisis can be viewed as a highly stressful event affecting a vast portion of the population. We found that, following the coronavirus outbreak, strong believers increased their commitment to religious beliefs while non-believers reported an increased scepticism towards religion. Moreover, the level of religiosity determined the relationship between coronavirus-related anxiety and change in religious beliefs: for strong believers anxiety strengthened religious beliefs, while for non-believers anxiety lowered these beliefs.

The relationship between religiosity and change in religious beliefs in response to the coronavirus pandemic can be interpreted in at least two ways. A first explanation focuses on inferential processes, assuming that people with different religiosity have interpreted differently the coronavirus outbreak. Religious people might interpret this theologically, for example as manifestation of God punishing or testing humans, or as expression of the devil’s intervention. Hence, occurrence of this event might reinforce religious beliefs. Conversely, non-believers might interpret the coronavirus outbreak as confirming God’s absence or uninterest in human affairs, thus weakening religious beliefs. A second explanation stresses the role of anxiety (Jong et al., 2012; Vail et al., 2010), for example proposing that death anxiety promotes commitment to the own prior belief system. Comparing inferential and anxiety-related explanations, both fit with our observation that the coronavirus outbreak leads strong believers to strengthen their religious beliefs and non-believers to weaken their beliefs. However, only anxiety-related accounts explain the observation that the relation between anxiety and change in religious beliefs depends on prior religiosity in such a way that, for strong believers, anxiety strengthen religious beliefs, while for non-believers anxiety weakens religious beliefs (Jong et al., 2012).

Our findings do not support the possibility that, during the coronavirus pandemic, stress or stress-induced anxiety enhance religious beliefs for both believers and non-believers (Vail et al., 2012). On the contrary, we found that religious beliefs were bolstered in strong believers but lowered in non-believers, and that anxiety strengthened religious beliefs in strong believers but promoted scepticism in non-believers. Moreover, we found no evidence of any relation between feeling of control about the coronavirus threat and change in religious beliefs. This does not fit with theories proposing that religious beliefs are bolstered by perceiving lack of control (Kay et al., 2010; Norris & Inglehart, 2004; Rigoli, 2020). Note that, in general, previous research provides mixed evidence in support of theories emphasising lack of control or anxiety as factors supporting religion for both believers and non-believers (Jong et al., 2018). With this regard, it is helpful to highlight the specific context of our study, because the context might determine which specific effects are engaged. First, the coronavirus crisis is characterised by a public nature (e.g., by concerning of the whole globe and by engaging the media), a severe threat for health and life, a concern for both the self and others, and an influence upon daily life (e.g., consider the government’s prescription to stay home). Second, the coronavirus pandemic was still ongoing during testing, whereas other studies have examined a time when a stressful episode was already over.

## Limitations

We highlight some important limitations of our study. First, our focus is on the USA and the UK and on Christian faith. Whether our results can be generalised to other countries and to other religions remains to be established. Second, participants’ religiosity was assessed via self-report, which is a method vulnerable to issues such as virtue signalling, social desirability, and memory biases (Stone et al., 1999; Jong et al., 2017; Van de Mortel 2008). To integrate our study, future research might consider exploring religiosity by recording behavioural measures such as church attendance and donations to religious institutions. However, a problem of this approach in the context of the coronavirus pandemic might be that, for many people, the pandemic has impaired the possibility to perform common behaviours such as going to church. Hence, behavioural measures might be poor indexes of psychological states in unusual circumstances such as the coronavirus pandemic. Another fruitful approach could be to assess religiosity through implicit psychological methods, which are less affected by the problems listed above (Jong et al., 2012, 2017; LaBouff et al., 2010). Another limitation is that our study focuses on a specific time: March 2020. This corresponds to the initial period of the crisis, when arguably the consequences of the pandemic (such as the unprecedented lockdown measures implemented by governments) might have appeared as more unexpected and shocking. Hence, the changes in religiosity observed here might reflect coping strategies adopted as an immediate reaction to the pandemic. Whether these strategies are maintained also in the long run, namely in a later phase of the pandemic, remains a question open for future research.

It is important also to stress the limitations linked with the sampling method: recruitment was carried out via an online system (Prolific) where some categories (e.g., young, highly educated, and technology-proficient individuals) might be overrepresented compared to their actual frequency in the population (also, number of participants for each gender and country was established a priori). Moreover, variables such as general health condition and experience of other stressful events were not measured, although they might have an impact on the results.

Our study is cross-sectional, meaning that it does not allow us to clarify the nature of the relationship between anxiety and change in religious beliefs observed here. One possibility is that anxiety acts upon religious beliefs, a possibility implicit in the anxiety-related explanations described above. However, the opposite might be true, and religious beliefs might act upon anxiety. According to this alternative possibility, for strong believers the coronavirus pandemic might first strengthen religious beliefs (e.g., by supporting the claim that God has chosen to punish humanity) and this in turn might boost anxiety. Similarly, for non-believers the coronavirus pandemic might first weaken religious beliefs (e.g., by supporting the hypothesis that God does not exist) and this in turn might boost anxiety. Further research is necessary to clarify this aspect.

## Conclusion

To summarize, we have explored the impact of the coronavirus pandemic upon religious beliefs. We found that, in response to the coronavirus threat, strong believers enhanced religious beliefs further while non-believers appeared as being even more sceptic about religion. Moreover, for strong believers anxiety about the coronavirus strengthened religious beliefs while for non-believers anxiety weakened religious beliefs. The implications of this study are twofold. First, our findings contribute to research investigating the impact of stress on religiosity, supporting the idea that, at least in some circumstances, stress and anxiety bolster the commitment to prior belief systems, namely the Christian faith for strong believers and sceptic belief systems for non-believers. Second, our study contributes to broaden our knowledge on the consequences of the coronavirus pandemic. In addition to its medical implications, the coronavirus crisis represents also a dramatic challenge for the psychology and culture of many communities; hence, shedding light on these aspects represents an important research endeavour.
